# Integrated Network Pharmacology Analysis and *In Vitro* Validation Revealed the Potential Active Components and Underlying Mechanistic Pathways of Herba Patriniae in Colorectal Cancer

**DOI:** 10.3390/molecules26196032

**Published:** 2021-10-05

**Authors:** Huihai Yang, Man-Kit Cheung, Grace Gar-Lee Yue, Ping-Chung Leung, Chun-Kwok Wong, Clara Bik-San Lau

**Affiliations:** 1 Institute of Chinese Medicine and State Key Laboratory of Research on Bioactivities and Clinical Applications of Medicinal Plants, The Chinese University of Hong Kong, Shatin, New Territories, Hong Kong; 1155155311@link.cuhk.edu.hk (H.Y.); graceyue@cuhk.edu.hk (G.G.-L.Y.); pingcleung@cuhk.edu.hk (P.-C.L.); ck-wong@cuhk.edu.hk (C.-K.W.); 2 Department of Surgery, The Chinese University of Hong Kong, Shatin, New Territories, Hong Kong; mkcheung@cuhk.edu.hk; 3 Department of Chemical Pathology, The Chinese University of Hong Kong, Shatin, New Territories, Hong Kong; 4 Li Dak Sum Yip Yio Chin R&D Centre for Chinese Medicine, The Chinese University of Hong Kong, Shatin, New Territories, Hong Kong

**Keywords:** Herba Patriniae, *Patrinia heterophylla*, network pharmacology, colorectal cancer, CRC, ErbB signaling pathway, FoxO signaling pathway

## Abstract

Herba Patriniae (HP) are medicinal plants commonly used in colorectal cancer (CRC) patients. In this study, network pharmacology was used to predict the active components and key signaling pathways of HP in CRC. *Patrinia heterophylla*, one type of HP, was chosen for validation of the network pharmacology analysis. The phytochemical profile of *Patrinia heterophylla* water extract (PHW) was determined by UHPLC-MS. MTT, RT-PCR, and Western blot assays were performed to evaluate the bioactivities of PHW in colon cancer cells. Results showed that 15 potentially active components of HP interacted with 28 putative targets of CRC in the compound–target network, of which asperglaucide had the highest degree. Furthermore, the ErbB signaling pathway was identified as the pathway mediated by HP with the most potential against CRC. Both RT-PCR and Western blot results showed that PHW significantly downregulated the mRNA and protein levels of EGFR, PI3K, and AKT in HCT116 cells. Asperglaucide, present in PHW, exhibited an anti-migratory effect in HCT116 cells, suggesting that it could be an active component of PHW in CRC treatment. In conclusion, this study has provided the first scientific evidence to support the use of PHW in CRC and paved the way for further research into the underlying mechanisms of PHW against CRC.

## 1. Introduction

Colorectal cancer (CRC) is the third most prevalent cancer in the world [1]. In 2020, 104,610 new CRC cases and 53,200 deaths were estimated, which account for about 10% of all new cancer cases and deaths worldwide. Moreover, the number of new CRC cases is estimated to increase to nearly 2.5 million in 2035 [2]. Genomic instability is an essential pathogenic mechanism leading to CRC, including KRAS, BRAF, PIK3CA, and TP53 gene mutations, while corresponding cell signaling pathways linked to the initiation, development, and progression of CRC have been reported [3]. For instance, mitogen-activated protein kinases (MAPKs) and phosphoinositide 3-kinase (PI3K) are both involved in the proliferation and survival of colorectal cancer cells [3]. The Wnt and TGF-β pathways also play crucial roles in the differentiation and growth of colorectal cancer cells [4,5]. To date, palliative chemotherapy and radiotherapy have been the main strategies for controlling tumor progression, whereas surgical intervention is adopted when the progression of CRC is uncontrollable [2]. Although the 5-year survival rate for CRC is around 64%, the number reduces to 12% for metastatic CRC [1]. Therefore, investigation is still required to further explore the effective approaches to medical intervention into CRC.

Herba Patriniae, also known as “Bai Jiang Cao” in Chinese, belongs to the Valerianaceae family. Herba Patriniae consists of around 20 *Patrinia* species that were firstly recorded in “Shen Nong’s Herbal Classic” as medicinal materials. They are widely distributed in Eastern Asia and North America [6]. Ten species, three sub-species, and two variant species of the genus *Patrinia* have been used as medicinal herbs, which are grown in the eastern, central, southern, and southwestern regions of China, such as *Patrinia scabiosaefolia*, *Patrinia villosa*, *Patrinia heterophylla*, *Patrinia rupestris*, and *Patrinia scabra* [7]. As a folk herbal remedy, Herba Patriniae has long been used for clearing heat, detoxification, dispelling blood stasis, and relieving pain [6]. Modern pharmacological studies have reported that Herba Patriniae possesses potent antioxidant, anti-inflammation, and sedation properties [6]. Phytochemistry research has revealed that Herba Patriniae is high in triterpenoid saponins and volatile constituents [6]. More importantly, increasing evidence has shown that Herba Patrinia ethanol extract (PE) can induce apoptosis in colorectal cancer cells and suppress tumor proliferation and angiogenesis in CRC [8,9,10]. Moreover, PE exhibits potent inhibition of the growth of 5-fluorouracil-resistant CRC cells [11]. In the clinic, Herba Patriniae is commonly prescribed in herbal formulae for CRC treatment by Chinese medicine practitioners. Although the common practice of consuming Chinese herbal medicines is using a decoction (water extract), very few research studies have been conducted on Herba Patriniae water extract [12]. Therefore, it is important to investigate the potential bioactive compounds and pharmacological mechanisms of Herba Patriniae water extract in CRC.

Based on the holistic and systematic characteristics of traditional Chinese medicine (TCM) theory, it is widely accepted that herbal medicines can have multiple pharmacological effects [13]. Network pharmacology is a relatively new approach for pharmacological mechanistic studies and drug development, especially in the field of TCM [14]. Network-based analysis is capable of describing complexities among biological systems, drugs, and diseases, which share an integral philosophy similar to that of TCM [14]. For example, network pharmacology has previously been used to decipher the molecular mechanisms behind the anti-metastatic effect of *Oldenlandia diffusa* on breast cancer [15] and the inhibitory effect of the classical formula Zuojinwan on CRC [16]. In the present study, we combined network pharmacology and phytochemical analysis to examine the potential molecular mechanisms of Herba Patriniae water extract in CRC, with an attempt to identify the putative targets and underlying signaling pathways involved.

## 2. Results

### 2.1. Network Pharmacology Analysis

#### 2.1.1. Screening of Potentially Active Compounds of Herba Patriniae

**Figure 1:**
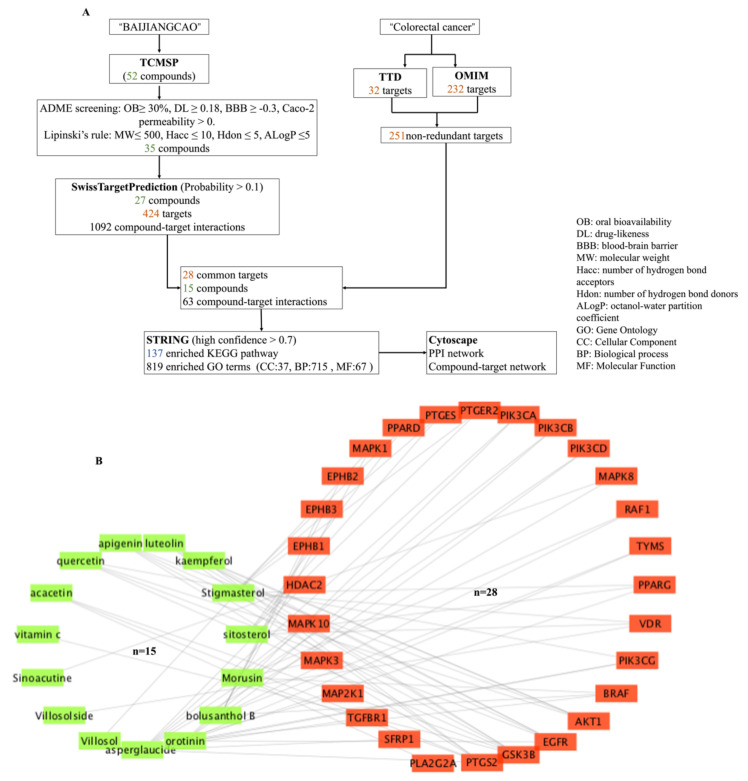
The flow chart of the screening process of network pharmacology (**A**) and compound–target interaction network of Herba Patriniae and CRC (**B**). The fifteen potentially active compounds of Herba Patriniae are in green, whereas the 28 CRC-related targets are in orange.

Based on the Traditional Chinese Medicine Systems Pharmacology Database and Analysis Platform (TCMSP), 52 compounds from Herba Patriniae were obtained, of which seven passed the “absorption, distribution, metabolism, and excretion” (ADME) screening criteria and 33 compounds fulfilled Lipinski’s rule (Figure 1A). A total of 35 non-redundant potentially active compounds of Herba Patriniae were obtained after removing duplicate compounds and those without a PubChem compound ID (CID) (Table 1).

#### 2.1.2. Putative Active Components and Targets of Herba Patriniae in CRC Treatment

A total of 424 predicted targets were obtained from 27 potentially active compounds of Herba Patriniae. In addition, 42 and 232 candidate targets of CRC were obtained from the Therapeutic Target Database (TTD) and the Online Mendelian Inheritance in Man (OMIM), respectively, comprising a total of 251 non-redundant targets. Twenty-eight of these targets were in common with those predicted from the 15 potentially active compounds of Herba Patriniae (Figure 1B).

The degree of a node in a network indicates the number of adjacent components [17]. As shown in Table 2, asperglaucide (degree = 10), orotinin (degree = 8), bolusanthol B (degree = 6), and morusin (degree = 6) were the compounds with the highest degrees in the compound–target interaction network, suggesting the major roles played by them in Herba Patriniae acting on CRC. Moreover, GSK3B (P49,841, degree = 6), PTGS2 (P35,354, degree = 6), EGFR (P00,533, degree = 5), and AKT1 (P31,749, degree = 4) were the target genes with the highest degrees in the network (Table 3).

#### 2.1.3. Protein–Protein Interaction (PPI) Analysis

**Figure 2:**
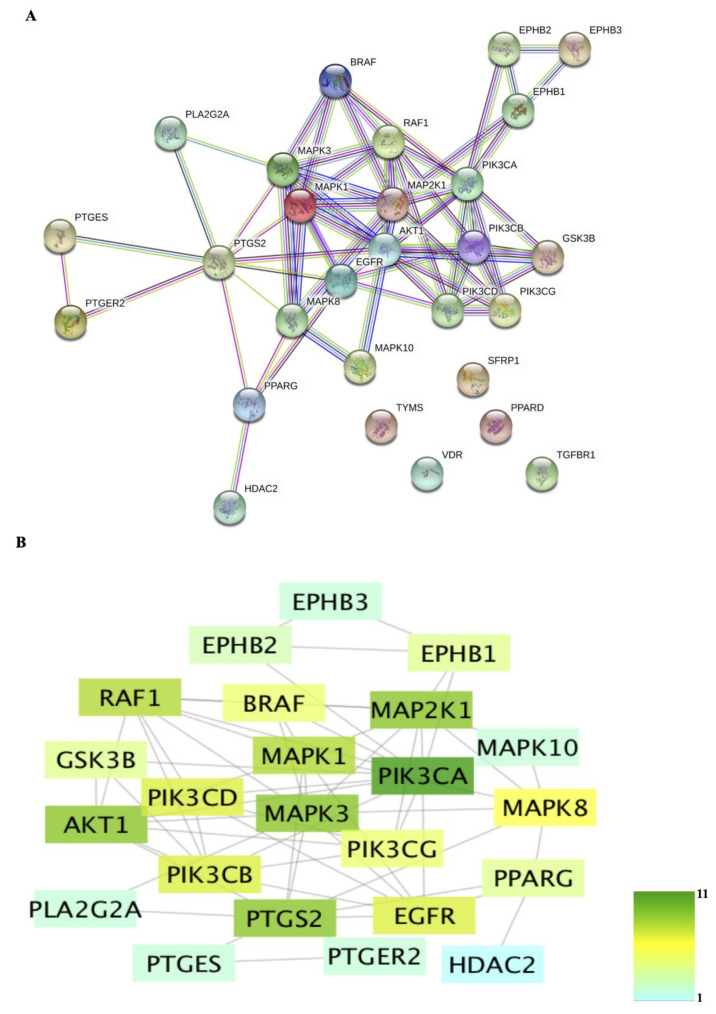
Protein–protein interaction (PPI) networks constructed in STRING (**A**) and Cytoscape (**B**). The nodes in panel B are colored according to their degree.

PPI networks were constructed in STRING and Cytoscape (Figure 2). The network constructed in STRING contained 28 nodes and 63 edges (Figure 2A), which is significantly more than the expected number of 16 edges (*P* < 1.0e^−16^). The PPI network constructed in Cytoscape contained 23 interconnected nodes (Figure 2B), with PIK3CA (degree = 11), AKT1 (degree = 9), MAP2K1 (degree = 9), MAPK3 (degree = 9), PTGS2 (degree = 9), MAPK1 (degree = 8), and RAF1 (degree = 8) having the highest degrees (Table 4).

#### 2.1.4. GO and KEGG Enrichment Analysis

**Figure 3:**
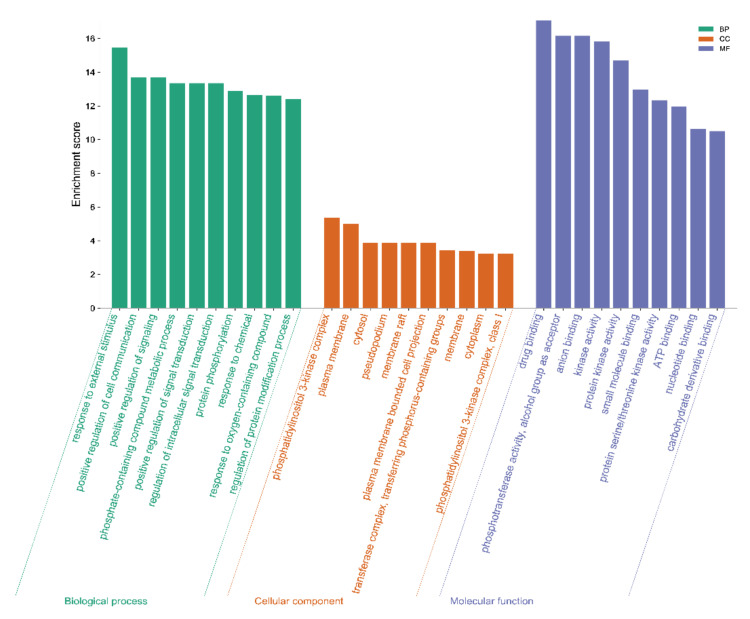
GO enrichment analysis. Top 10 significantly enriched GO terms in “biological process” (BP), “cellular component” (CC), and “molecular function” (MF) are shown. Enrichment scores represent −log *P* values.

**Figure 4:**
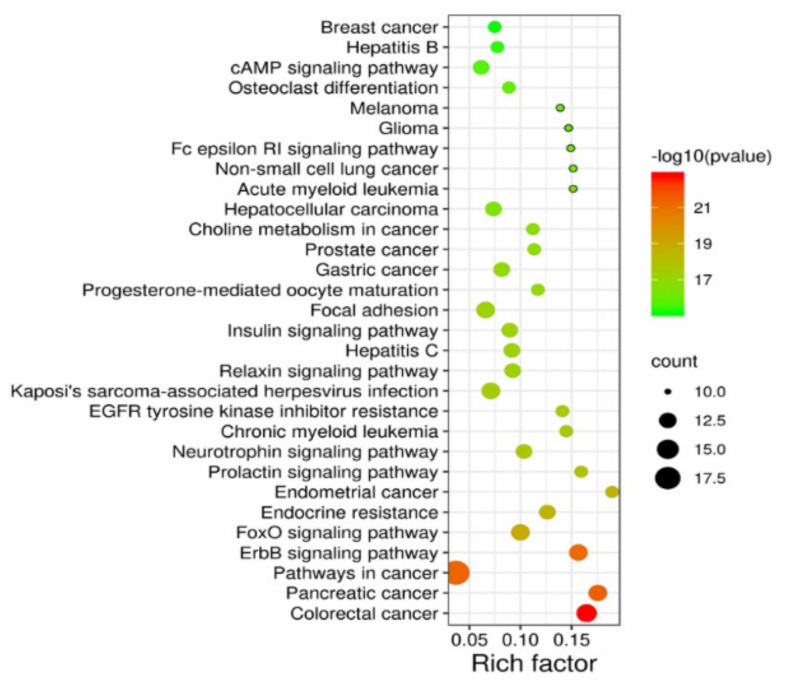
Bubble graph of the KEGG enrichment pathway analysis. The rich factor represents the ratio of the number of enriched genes in a KEGG category to the total number of genes in that category.

According to the analysis in STRING, a total of 819 GO enrichment results were obtained, including 715 terms in “biological process”, 67 terms in “molecular function”, and 37 terms in “cellular component”. The top 10 enriched GO terms in each category are shown in Figure 3. The screened targets were mainly involved in biological processes, including “response to external stimulus”, “positive regulation of cell communication”, “positive regulation of signaling”, and “phosphate-containing compound metabolic process”. While “drug binding” and “phosphotransferase activity” ranked the highest in the molecular function category, “phosphatidylinositol 3-kinase complex”, “plasma membrane”, and “cytosol” were the primary enriched cellular component categories. In addition, the 28 putative targets of the 15 potentially active components of Herba Patriniae were mapped onto 137 KEGG pathways. The top 30 enriched pathways are shown in Figure 4. The top five pathways with the lowest *P* values were related to colorectal cancer (hsa05210, count = 14), pancreatic cancer (hsa05212, count = 13), pathways in cancer (hsa05200, count = 19), the ErbB signaling pathway (hsa04012, count = 13), and the FoxO signaling pathway (hsa04068, count = 13).

### 2.2. The In Vitro Validation of the Network Pharmacology Analysis

*Patrinia heterophylla*, which is a species of Herba Patriniae commonly available in herbal markets in Hong Kong, was chosen for further in vitro validation on the molecular targets predicted by the above-described network pharmacology analysis.

#### 2.2.1. *Patrinia heterophylla* Water Extract (PHW) Exhibited Cytotoxicity on Colon Cancer Cell Lines

According to the traditional custom of boiling Chinese herbal medicines in water, the water extract of *Patrinia heterophylla* was used in our study. Cell viability after PHW treatment was assessed by MTT assay. Results show that PHW exhibited significant cytotoxicities on various human colon cancer cell lines. As shown in Table 5, HCT116 cells were the most sensitive to PHW treatment, with IC_50_ values of 638.1 ± 52.0 μg/mL and 378.1 ± 19.5 μg/mL after 24 h and 48 h of treatment, respectively. However, there was no cytotoxic activity towards normal human skin fibroblast cells (Hs27) at doses of 0–800 μg/mL, suggesting its selective cytotoxicity towards colon cancer cells. The IC_50_ values of 5-fluorouracil, which was used as a positive control drug, on these cell lines are also shown in Table 5.

#### 2.2.2. The Effects of *Patrinia heterophylla* Water Extract (PHW) on the mRNA and Protein Expression of EGFR, PI3K (PIK3CA), and AKT (AKT1) in HCT116 Cells

**Figure 5:**
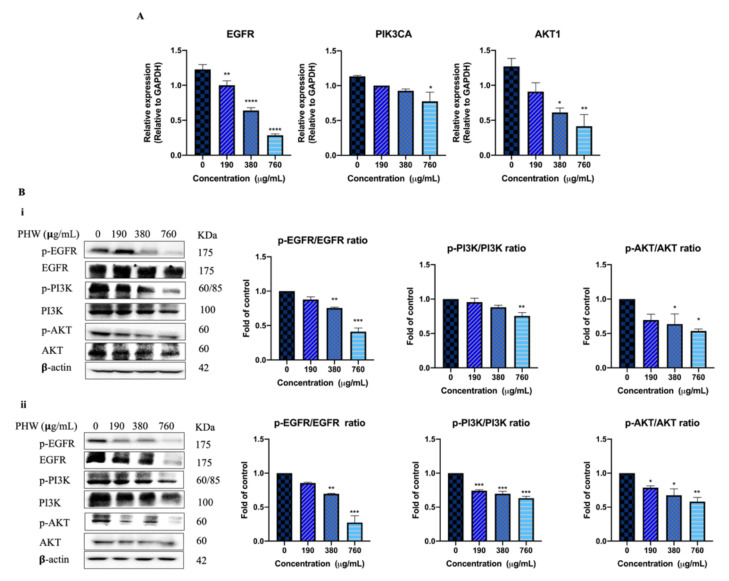
The effects of PHW on mRNA and protein expression in HCT116 cells. (**A**) RT-PCR results and (**B**) Western blot results after 24 h (i) and 48 h (ii) of treatment. Data are presented as the mean ± SD from at least three independent experiments. Compared with vehicle control, ^*^ p < 0.05, ^**^ p < 0.01, ^***^ p  < 0.001, ^****^ p < 0.0001.

The effects of PHW on the mRNA expression of EGFR, PIK3CA, and AKT1 were determined by real-time polymerase chain reaction (RT-PCR) analysis in HCT116 cells. The results show that the mRNA expression of EGFR, PIK3CA, and AKT1 was downregulated after 24 h of PHW treatment (190–760 μg/mL) in a concentration-dependent manner compared with the untreated group (Figure 5A). To further confirm the RT-PCR results, the expression of EGFR, PI3K, and AKT was assessed by the Western blot method. As shown in Figure 5B, compared with the untreated group, PHW significantly decreased the values of p-EGFR/EGFR, p-PI3K/PI3K, and p-AKT/AKT after 24 h and 48 h of treatment in a dose-dependent manner in HCT116 cells, which indicates that PHW could inhibit the EGFR/PI3K/AKT signaling pathway in HCT116 cells.

### 2.3. Identification of Chemical Compounds in Patrinia heterophylla Water Extract (PHW)

To further confirm the presence of active components in *Patrinia heterophylla* water extract (PHW), the chemical profiles of PHW were analyzed by UHPLC-MS. The yield of PHW was 12.2% *w*/*w*. Based on the above results from the Traditional Chinese Medicine Systems Pharmacology Database and Analysis Platform (TCMSP), only four compounds (asperglaucide, villosol, villosolside, and ascorbic acid (vitamin C)) were tentatively identified to be present in PHW using UHPLC-MS (Supplementary Figure S1, Table S1). The content of asperglaucide in PHW is 0.78 mg/100 g dry extract as detected by the LCMS method. Only the amount of asperglaucide was quantified as it had the highest score among these four compounds in the compound–target interaction network.

### 2.4. The Potential Effects of Asperglaucide on Colon Cancer Cells

**Figure 6:**
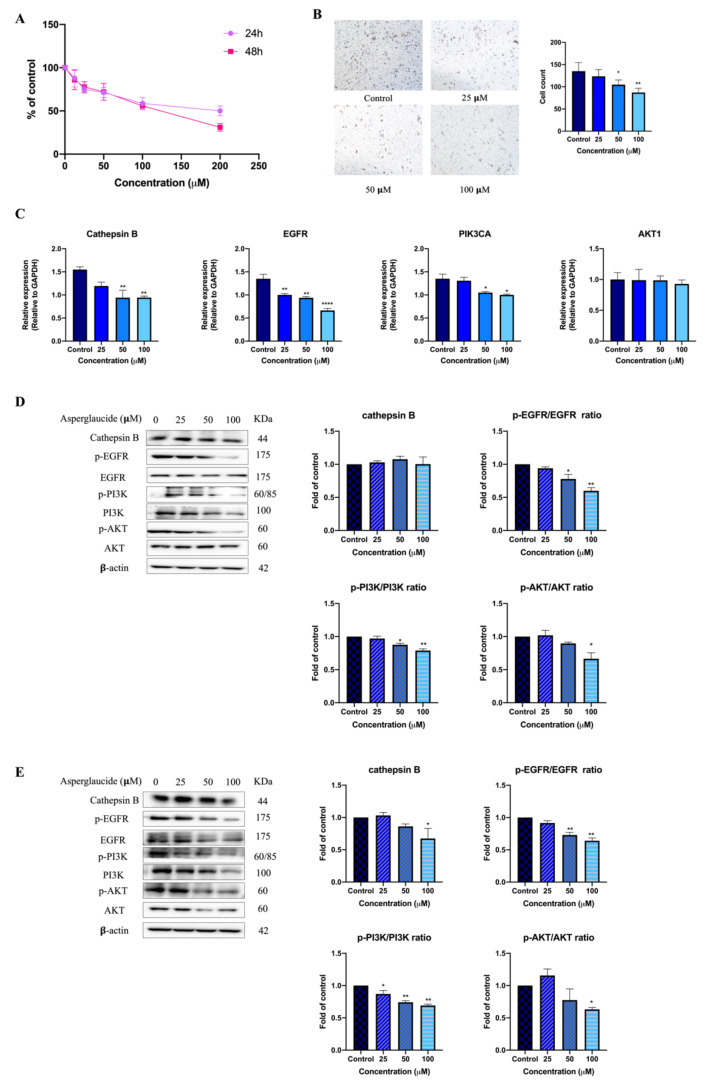
The effects of asperglaucide on cytotoxicity, cell migration, and mRNA expression in HCT116 cells. (**A**) MTT results; (**B**) Transwell assay results; (**C**) RT-PCR results. (**D**) Western blot results after 24 h of treatment; (**E**) Western blot results after 48 h of treatment. Data are presented as the mean ± SD from at least three independent experiments. Compared with vehicle control, ^*^ p < 0.05, ^**^ p < 0.01, ^****^ p < 0.0001.

Asperglaucide (aurantiamide acetate) was found to be a cathepsin L and cathepsin B inhibitor in a proteinase inhibitory experiment [18]. In the present study, asperglaucide not only exhibited the highest score in the compound–target interaction network, but it was also detected in *Patrinia heterophylla* water extract by UHPLC assay. Thus, it is meaningful to explore its potential activities in colon cancer cells. As shown in the MTT results, asperglaucide exhibited cytotoxic activities on HCT116 cells with IC_50_ values of 188.7 ± 7.21 μM and 107.7 ± 11.02 μM after 24 h and 48 h of treatment, respectively (Figure 6A). Moreover, asperglaucide at 50 and 100 μM possessed an inhibitory effect on cell migration in HCT116 cells (Figure 6B). Furthermore, asperglaucide at greater than 25 μM significantly decreased the mRNA levels of cathepsin B, EGFR, and PIK3CA (Figure 6C). Western blot results further indicate that asperglaucide can inhibit the levels of p-EGFR/EGFR, p-PI3K/PI3K, and p-AKT/AKT after 24 h and 48 h of treatment, while the expression of cathepsin B statistically significantly decreased after 48 h of treatment with asperglaucide at 100 μM.

## 3. Discussion

The concept of network pharmacology was established in 2007 [19]. With the help of this approach, many complicated and complex therapeutic mechanisms of TCM prescriptions have been successfully elucidated [20]. Herba Patriniae, comprised of various *Patrinia* species, has been used in multiple herbal medicine formulae to treat enteric diseases, including CRC [7]. However, there is very little scientific evidence on the therapeutic mechanisms of Herba Patriniae in CRC; in particular, scientific evidence on the commonly used species *Patrinia heterophylla* water extract (PHW) in CRC is lacking. Hence, in this study, we made an attempt to use network pharmacology to explore and predict the potential active components and underlying mechanisms of PHW in treating CRC and validated the analysis by in vitro functional assays and examining the changes in mRNA and protein expression of the target molecules in colon cancer cells.

The PPI network is a major principle of biological organization that demonstrates the importance of fundamental cellular processes [21]. Abnormality of a protein in a PPI network can produce a series of functional abnormalities, leading to the occurrence of diseases, including cancer [17]. PIK3CA, AKT1, MAP2K1, MAPK3, and PTGS2 were found to be the top five targets in the PPI network constructed in the present study. PIK3CA can interact with AKT1, RAF1, EGFR, MAP2K1, PIK3CG, EPHB2, EPHB1, PIK3CB, PIK3CD, GSK3B, and BRAF. Mutations in PIK3CA can activate the PI3K signaling pathway and downstream AKT signaling, leading to increased proliferation and invasion of tumor cells and increased metastasis [22]. AKT1 is one of the isoforms of the AKT family, which is related to the survival, invasion, and metastasis of cancer cells [23]. Both MAP2K1 and MAPK3 encode proteins that are involved in the MAPK signaling pathway, which affects many cellular processes, including proliferation, differentiation, transcription, and development [24]. PTGS2 is a key enzyme mediating the neosynthesis of prostaglandin, which was found to be expressed in epithelial cells in CRC [25]. Overall, these genes may play important roles in the anti-cancer activity of Herba Patriniae in CRC based on the network pharmacological analysis.

**Figure 7:**
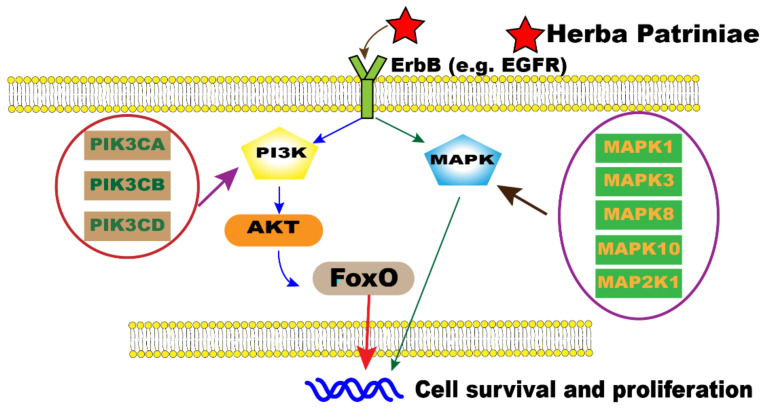
Schematic representation of the potential molecular machinery behind the anti-cancer activity of Herba Patriniae in CRC via the ErbB and FoxO signaling pathways. EGFR, epidermal growth factor receptor; PI3K, phosphoinositide-3 kinases; PIK3CA, phosphatidylinositol-4,5-bisphosphate-3 kinase catalytic subunit alpha; PIK3CB, phosphatidylinositol-4,5-bisphosphate-3 kinase catalytic subunit beta; PIK3CD, phosphatidylinositol-4,5-bisphosphate-3 kinase catalytic subunit delta; MAPK, mitogen-activated protein kinase; MAPK1, mitogen-activated protein kinase 1 (ERK2); MAPK3, mitogen-activated protein kinase 3 (ERK 1); MAPK8, mitogen-activated protein kinase 8 (JNK 1); MAPK10, mitogen-activated protein kinase 10 (JNK 3); MAP2K1, mitogen-activated protein kinase kinase 1 (MEK1); AKT, protein kinase B; FoxO, forkhead box protein O.

In our study, the ErbB and FoxO signaling pathways were found to be significantly enriched in the KEGG enrichment analysis. In fact, ErbB family members are overexpressed or mutated in many human cancers, including CRC [26]. They are involved in controlling the growth, survival, and metastasis of CRC [27]. The ErbB signaling pathway consists of receptor tyrosine kinases such as EGFR, HER2, HER3, and HER4 [28]. EGFR is located upstream of the MAPK pathway, which is regulated by the MAP2K1, MAPK1, MAPK10, MAPK3, and MAPK8 genes [29]. Moreover, EGFR can activate the PI3K/AKT pathway in human cancer [30]. It was reported that laminarin isolated from marine brown algae could induce apoptosis of HT-29 cells via regulation of ErbB signaling [31]. Allicin was also reported to suppress the growth and metastasis of gastric carcinoma cells via the inhibition of ErbB signaling [32]. On the other hand, FoxO is one of the forkhead transcription factor subfamilies and plays a pivotal functional role in cellular differentiation, proliferation, and apoptosis in multiple cancers [33]. FoxO family proteins are downstream of AKT and are triggered by the PI3K/AKT pathway, which mediates cell proliferation and growth [33]. It was reported that luteolin exerts cytotoxicity on human colon cancer cells via mediating the ERK/FoxO signaling pathway [34]. Collectively, the network pharmacology analysis results suggest that Herba Patriniae may play roles in the suppression of CRC via mediation of the ErbB and FoxO signaling pathways (Figure 7).

Further experiments, including UHPLC-MS analysis of PHW as well as an MTT assay, RT-PCR, and Western blot analysis, on colon cancer cells were performed to validate the network pharmacological results. An MTT assay is commonly used to evaluate the cytotoxic potential of a drug [35]. In this study, we showed for the first time that PHW could exhibit cytotoxic effects on human colonic cancer cells after 24 h and 48 h of treatment by an MTT assay. In addition, RT-PCR and a Western blot analysis were used for assessing the expression of mRNA and proteins that are related to the ErbB signaling pathway. RT-PCR results indicate that PHW could significantly decrease the mRNA expression of EGFR, PIK3CA, and AKT1 in HCT116 cells. Western blot results further prove that PHW could inhibit the expression of key proteins of the ErbB signaling pathway in HCT116 cells. However, the effect of PHW on the MAPK signaling pathway will need to be investigated in the future. On the other hand, UHPLC-MS analysis and the characteristic fragmentation revealed that only four compounds reported in the compound–target interaction network (Table 2) could be detected in PHW, suggesting that the other compounds may not be water soluble. Among these four compounds detected in PHW, it was previously shown that villosol, villosolside, and ascorbic acid (vitamin C) have anti-inflammatory effects on lipopolysaccharide-induced RAW 264.7 cells [36]. Asperglaucide (aurantiamide acetate) was reported to exhibit anti-inflammatory and anti-viral activities in influenza A virus-infected cells [37]. In addition, asperglaucide isolated from *Clematis terniflora* could suppress the growth of gliomas via inhibiting autophagic flux [38]. It is important to note that asperglaucide has previously been reported to be a cathepsin inhibitor, in particular cathepsin L and cathepsin B, with an IC_50_ of 12 μM and 49 μM, respectively [18]. In fact, it has previously been reported that aberrant overexpression of cathepsin B is a significant factor in the development, invasion, and metastasis of CRC [39]. Herein, asperglaucide not only exhibited the highest degree in our compound–target interaction network, but it also suppressed cell migration and decreased the expression of cathepsin B in HCT116 cells (Figure 6). As shown in Figure 1, asperglaucide putatively regulates PIK3CA gene expression according to the network pharmacology analysis. Our RT-PCR and Western blot results further confirm that asperglaucide significantly downregulated the expression of EGFR, PI3K (PIK3CA), and AKT in HCT116 cells. Taken together, these results suggest that asperglaucide could be an active component in PHW against CRC, but whether it is the major active component will need further experimental confirmation.

In conclusion, network pharmacology is a useful tool for predicting active components of herbal medicines and their underlying molecular mechanisms of action. Our study has provided the first pieces of scientific evidence to support the use of Herba Patriniae, specifically *Patrinia heterophylla* water extract, in CRC treatment, with asperglaucide being the potential active component. Our results certainly pave the way for further research into the mechanisms of action of PHW in CRC treatment.

## 4. Materials and Methods

### 4.1. Search for Potentially Active Compounds in Herba Patriniae

Information on all reported chemical components of Herba Patriniae was collected from the Traditional Chinese Medicine Systems Pharmacology Database and Analysis Platform (TCMSP, https://tcmspw.com/tcmsp.php, accessed on 10 August 2021) using the keyword “Baijiangcao”. The retrieved compounds were then screened by ADME parameters and Lipinski’s rule. The ADME parameters include oral bioavailability (OB) ≥ 30%, drug likeness (DL) ≥ 0.18, blood–brain barrier (BBB) ≥ −0.3, and Caco-2 permeability > 0 [17]. Lipinski’s rule includes molecular weight (MW) ≤ 500, number of hydrogen bond acceptors (Hacc) ≤ 10, number of hydrogen bond donors (Hdon) ≤ 5, and octanol–water partition coefficient (LogP) ≤ 5 [40]. Components that did not satisfy either the ADME parameters or Lipinski’s rule were excluded.

### 4.2. Prediction of Putative Targets of Herba Patriniae and CRC

SMILE structures of the screened compounds of Herba Patriniae were obtained from PubChem (http://pubchem.ncbi.nlm.nih.gov/, accessed on 10 August 2021) [41] and used as inputs into target prediction in the SwissTargetPrediction database (http://www.swisstargetprediction.ch/, accessed on 10 August 2021) [42]. Target genes of CRC were collected using the keyword “colorectal cancer” from the Therapeutic Target Database (TTD, http://db.idrblab.org/ttd/, accessed on 10 August 2021) [43] and the Online Mendelian Inheritance in Man (OMIM, https://omim.org/, accessed on 10 August 2021) [44]. Common targets between Herba Patriniae components and CRC were then identified.

### 4.3. Network Construction and Functional Enrichment Analysis

Compound–target and protein–protein interaction (PPI) networks were constructed and analyzed in Cytoscape v3.8.0. The results of PPI in the human genome and significantly enriched Gene Ontology (GO) terms and the Kyoto Encyclopedia of Genes and Genomes (KEGG) pathways with a false discovery rate < 0.05 were acquired from STRING v11 (http://string-db.org/, accessed on 10 August 2021) [45]. Only high-confidence interactions with scores > 0.7 were kept. For each network, the topological properties “degree”, “betweenness centrality”, “closeness centrality”, and “average shortest path length” were calculated using the NetworkAnalyzer plugin [46] to screen for putative nodes of topological importance. In general, the higher a node’s degree, betweenness centrality, or closeness centrality, the more important that node is in the network [47]. The enriched GO terms and KEGG pathways were visualized using tools on the bioinformatics web server (http://bioinformatics.com.cn/, accessed on 10 August 2021).

### 4.4. Plant Materials and Extract Preparation

The plant material *Patrinia heterophylla* Bunge, being the commonly used *Patrinia* species in Hong Kong, was purchased from Zisun Pharmaceutical Company Limited (Lot No. 190,701, Hong Kong, China) and morphologically authenticated by the botanist Dr. David Tai-Wai Lau of the Shiu-Ying Hu Herbarium of The Chinese University of Hong Kong. A voucher specimen (No. 3656) was kept at the museum of the Institute of Chinese Medicine at The Chinese University of Hong Kong. Dried plant material was powdered by a pulverizer, and 30 g of the powder was soaked in distilled water (at a solid-to-liquid ratio of 1:10) for 1 h at room temperature. The solution was then heated at 100 °C for 1 h. After cooling, the extract was filtered using filter paper, and then the same volume of distilled water was added into the container and the extraction was repeated once. The solution collected was concentrated to 300 mL by using a rotary evaporator (EYELA, Chui-Ku, Tokyo, Japan) and then lyophilized using a freeze dryer (Labconco, Kabsas City, MO, USA).

### 4.5. Chemical Pofiling of Patrinia heterophylla Water Extract (PHW) Using UHPLC-MS

For ultra-high-pressure liquid chromatography coupled with mass spectrometry (UHPLC-MS) analysis, 20 mg of dried *Patrinia heterophylla* water extract (PHW) was redissolved in 1 mL of deionized water and filtered through a 0.2 μm polytetrafluoroethylene membrane filter. Five microliters of the solution was then injected into an Agilent 1290 UHPLC with a 6530 QTOF system (Agilent, Santa Clara, CA, USA). The column used was an Agilent ZORBAX Eclipse Plus C18 RRHD (1.8 μm, 3 mm × 100 mm) with a guard column. Chromatographic separation was conducted at 40 °C under gradient conditions at a flow rate of 0.5 mL/min. The LC system was as follows: mobile phase: (A) 0.1% formic acid in deionized distilled water, and (B) 0.1% formic acid in acetonitrile; gradient: 0–1 min, 5% B; 1–10 min, 5–37% B; 10–11 min, 37–100% B; 11–13 min, 100% B. The column was re-equilibrated for 2 min after each injection. High-purity nitrogen was used as the curtain and collision gas with a flow rate of 10 L/min. The drying gas temperature was set at 350 °C, and the nebulizer pressure was set at 50 psi. Spectra were recorded in both positive mode and negative mode at a spray voltage of 4000 V. The mass scan range was set between 50 and 1100. Data analysis was performed using Agilent MassHunter Workstation Qualitative Analysis Software (Agilent, Santa Clara, CA, USA, version B.07.00).

### 4.6. UHPLC-MS Analysis for the Quantification of Asperglaucide

The analysis was conducted using an Agilent 1290 UHPLC with a 6530 QTOF system (Agilent, Santa Clara, CA, USA). The column used was an Agilent ZORBAX Eclipse Plus C18 RRHD (1.8 µm, 3.0 mm × 100 mm) with a guard column. The chromatographic separation was conducted at 40 °C under isocratic elution of 0.1% formic acid in deionized water (A): 0.1% formic acid in acetonitrile (B) (48:52) at a flow rate of 0.5 mL/min for 5 min. The column was flushed with 100% B for 3 min and re-equilibrated for another 2 min after each injection. High-purity nitrogen was used as the curtain and collision gas with a flow rate of 10 L/min. The drying gas temperature was set at 350 °C, and the nebulizer pressure was set at 50 psi. Spectra were recorded in positive ion mode at a spray voltage of 4000 V. The mass scan range was set between 50 and 950 *m*/*z*. Data analysis was performed using Agilent MassHunter Workstation Qualitative Analysis Software (Agilent, Santa Clara, CA, USA, version B.07.00). Asperglaucide was determined at 445.2202 *m*/*z* [M + H]^+^.

### 4.7. Cell Culture

The human colon adenocarcinoma cell lines HCT116, HT29, and LoVo, as well as the human normal skin fibroblast cell line Hs27, were obtained from ATCC (American Type Culture Collection, Manassas, VA, USA). HCT116 and HT29 cells were cultured in McCoy’s 5A medium with 10% (*v*/*v*) fetal bovine serum (FBS) and 1% (*v*/*v*) penicillin/streptomycin, whereas LoVo and Hs27 cells were cultured in Dulbecco’s Modified Eagle’s medium. All cell culture media and supplements were purchased from Thermo Fisher Scientific (Waltham, MA, USA). All cells were incubated at 37 °C with 5% CO_2_ in an incubator (BINDER, Tuttlingen, Germany).

### 4.8. MTT Assays

The viability of colon cancer cells and normal cells after PHW treatment was tested using a 3-(4,5-dimethylthiazol-2-yl)-2,5-diphenyltetrazolium bromide (MTT) assay. In brief, 5×10^3^ cells per well were seeded in a 96-well microplate and incubated overnight. The cells were then added to 100 μL of various doses of PHW (0–800 μg/mL) and incubated for 24 h or 48 h. Thirty microliters of MTT solution (5 mg/mL) was then added to each well and the cells were incubated for another 4 h. MTT purple crystals were dissolved in dimethyl sulfoxide and the optical density at 540 nm (OD_540_) was detected using a μQuant microplate spectrophotometer (Biotek, Vermont, VT, USA).

### 4.9. Transwell Migration Assay

The migratory effect on colon cancer cells was evaluated by a transwell migration assay. Briefly, cells (5×10^4^ in 100 μL) with same volume of serum-free medium containing asperglaucide (0–100 μM) were placed into the upper chamber of a transwell (Corning, New York, NY, USA), while 500 μL of medium with 10% FBS (used as a chemoattractant medium) was added into the lower chamber. After 18 h of incubation, the cells were fixed with methanol for 3 min and then stained with hematoxylin for 5 min. The cells on the top surface of the membrane were scraped away using a cotton bud, and then the remaining cells that had adhered to the underside of the membrane were photographed using an Olympus IX-71 microscope with a digital camera (Olympus, Tokyo, Japan). The number of migrated cells was counted using Image J software, which reflected the migration ability.

### 4.10. Real Time Quantitative PCR (RT-PCR) Analysis

HCT116 (5×10^5^) cells were seeded in 6-well plates and incubated overnight. Fresh medium with PHW (190–760 μg/mL) or asperglaucide (purity ≥ 98%, ChemFaces, China) (25–100 μM) was added into the plates and the cells were treated for 24 h. Total RNA from the HCT116 cells was then extracted by 1 mL of Trizol reagent according to the manufacturer’s protocol (Invitrogen, Waltham, MA, USA). The primer sequences were synthesized by Invitrogen (Waltham, MA, USA) and are listed in Table S2. The target gene mRNA levels were normalized to the GAPDH (as an internal control) mRNA levels and then expressed using the 2^−ΔΔCt^ method.

### 4.11. Western Blot Analysis

HCT116 (1×10^6^) cells were seeded in 100 mm^2^ culture dishes overnight. Fresh medium with PHW (190–760 μg/mL) was added into the dishes and the cells were treated for 24 h and 48 h. Cells were collected after washing with cold PBS and scraping. Cells were lysed with lysis buffer (Beyotime Institute of Biotechnology, Shanghai, China) on ice. The concentration of protein was quantified using a BCA kit (Thermo Fisher Scientific, Walham, MA, USA). An equivalent amount of protein was loaded on 10% SDS-PAGE gels and then transferred to polyvinylidene fluoride (PVDF) membranes. The membranes were blocked with 5% non-fat milk for an hour, and then incubated with primary antibodies (1:1000) at 4 °C overnight. The membranes were then washed three times (15 min each time) with TBS-T solution and incubated with secondary antibodies (1:3000) for 1 h. After three washes with TBS-T (15 min each time), the blots were detected using an ECL kit (GE Healthcare Life Sciences, Marlborough, MA, USA) and photographed using ChemiDoc XRS + Imaging Systems (Bio-Rad, Hercules, CA, USA). The bands were quantified by ImageJ (NIH, Bethesda, MD, USA). The primary antibodies (EGFR, p-EGFR, PI3K, p-PI3K, AKT, p-AKT, and cathepsin B) were purchased from Cell Signaling Technology (Danvers, MA, USA) and β-actin was purchased from Sigma-Aldrich (St. Louis, MO, USA). The horseradish peroxidase secondary antibodies were purchased from Akoya Biosciences (Marlborough, MA, USA). Information on the antibodies, including catalog numbers and dilutions, is shown in Table S3. The intensities of bands were normalized to their own internal standard proteins (β-actin) for each protein sample. The quantitative data are presented as fold of untreated control.

### 4.12. Statistical Analysis

Data from the cell culture experiments are expressed as the mean ± standard deviation (SD). Differences among groups were tested using one-way ANOVA. *p* values < 0.05 were considered statistically significant. Statistical analysis was performed using GraphPad Prism v 8.0 software (GraphPad Software, San Diego, CA, USA).

## Figures and Tables

**Table 1:** Thirty-five potentially active compounds of Herba Patriniae according to ADME parameters and Lipinski’s rule.

PubChem CID	Mol ID	Compound Names	SMILES	MW	AlogP	Hdon	Hacc	OB (%)	Caco-2	BBB	DL
821366	MOL001697	Sinoacutine	CN1CCC23C=C(C(=O)C=C2C1CC4=C3C(=C(C=C4)OC)O)OC	325.39	1.00	1.00	5.00	63.39	0.72	0.36	0.53
5280442	MOL001689	Acacetin	COC1=CC=C(C=C1)C2=CC(=O)C3=C(C=C(C=C3O2)O)O	284.28	2.59	2.00	5.00	34.97	0.67	−0.05	0.24
10594416	MOL001678	Bolusanthol B	CC(=CCC1=C(C(=CC(=C1)C2COC3=CC(=CC(=C3C2=O)O)O)O)O)C	356.40	3.77	4.00	6.00	39.94	0.29	−0.35	0.41
13855373	MOL001677	Asperglaucide	CC(=O)OCC(CC1=CC=CC=C1)NC(=O)C(CC2=CC=CC=C2)NC(=O)C3=CC=CC=C3	444.57	4.02	2.00	6.00	58.02	0.28	−0.22	0.52
20055981	MOL001676	Vilmorrianine C	CCN1CC2(CCC(C34C2C(C(C31)C5(CC(C6CC4C5C6OC(=O)C7=CC=C(C=C7)OC)OC)OC(=O)C)OC)OC)COC	627.85	1.55	0	10.00	33.96	0.59	0.14	0.22
5280794	MOL000449	Stigmasterol	CCC(C=CC(C)C1CCC2C1(CCC3C2CC=C4C3(CCC(C4)O)C)C)C(C)C	412.77	7.64	1.00	1.00	43.83	1.44	1.00	0.76
5280863	MOL000422	Kaempferol	C1=CC(=CC=C1C2=C(C(=O)C3=C(C=C(C=C3O2)O)O)O)O	286.25	1.77	4.00	6.00	41.88	0.26	−0.55	0.24
222284	MOL000359	Sitosterol	CCC(CCC(C)C1CCC2C1(CCC3C2CC=C4C3(CCC(C4)O)C)C)C(C)C	414.79	8.08	1.00	1.00	36.91	1.32	0.87	0.75
5280343	MOL000098	Quercetin	C1=CC(=C(C=C1C2=C(C(=O)C3=C(C=C(C=C3O2)O)O)O)O)O	302.25	1.50	5.00	7.00	46.43	0.05	−0.77	0.28
5280445	MOL000006	Luteolin	C1=CC(=C(C=C1C2=CC(=O)C3=C(C=C(C=C3O2)O)O)O)O	286.25	2.07	4.00	6.00	36.16	0.19	−0.84	0.25
2758	MOL000122	1,8-cineole	CC1(C2CCC(O1)(CC2)C)C	154.28	2.15	0	1.00	39.73	1.57	2.06	0.05
2724159	MOL001254	(S)-p-Mentha-1,8-dien-7-al	CC(=C)C1CCC(=CC1)C=O	150.24	2.67	0	1.00	39.00	1.36	1.57	0.03
159055	MOL000130	D-Camphor	CC1(C2CCC1(C(=O)C2)C)C	152.26	1.94	0	1.00	67.17	1.29	1.71	0.05
8106	MOL001672	Hexylthiol	CCCCCCS	118.27	2.85	0	0	37.37	1.54	1.67	0
74346	MOL001673	2-Ethyl-5-methylfuran	CCC1=CC=C(O1)C	110.17	1.89	0	1.00	48.78	1.68	2.02	0.01
127454	MOL001674	Villosolside	CC1C2CC(C(C2COC1=O)(C)OC3C(C(C(C(O3)CO)O)O)O)O	362.42	−1.89	5.00	9.00	14.44	−1.51	−2.52	0.35
5490819	MOL001675	Villosol	CC(=C)C1CC2=C(O1)C=C(C3=C2OC4=C(C3=O)C5=CC(=C(C=C5OC4)OC)OC)O	200.26	−0.14	2.00	4.00	99.30	−0.17	−0.74	0.08
6432452	MOL001679	(1*S*,4*S*)-1-Isopropyl-4-methyl-3-bicyclo[3.1.0]hexanone	CC1C2CC2(CC1=O)C(C)C	152.26	1.77	0	1.00	75.17	1.35	1.76	0.05
87691	MOL001680	Loganin	CC1C(CC2C1C(OC=C2C(=O)OC)OC3C(C(C(C(O3)CO)O)O)O)O	390.43	−2.08	5.00	10.00	5.90	−1.48	−2.26	0.44
10466307	MOL001681	Methyl (1*R*,4a*S*,6*S*,7*R*,7a*S*)-1,6-dihydroxy-7-methyl-1,4a,5,6,7,7a-hexahydrocyclopenta[d]pyran-4-carboxylate	CC1C(CC2C1C(OC=C2C(=O)OC)O)O	228.27	−0.33	2.00	5.00	29.99	−0.36	−1.05	0.10
588991	MOL001684	6-Isoquinolylamine	C1=CC2=C(C=CN=C2)C=C1N	144.19	0.84	2.00	2.00	30.46	0.67	0.11	0.04
21721831	MOL001685	Orotinin	CC(=CCC1=C2C(=C(C3=C1OC(CC3=O)C4=C(C=CC=C4O)O)O)C=CC(O2)(C)C)C	422.51	4.94	3.00	6.00	1.01	0.64	0.02	0.76
3059326	MOL001687	Patrinoside-aglycone	CC(C)CC(=O)OC1C2C(CC(C2CO)O)C(=CO1)CO	300.39	−0.26	3.00	6.00	14.06	−0.65	−0.98	0.18
54670067	MOL001691	Vitamin C	C(C(C1C(=C(C(=O)O1)O)O)O)O	176.14	−1.76	4.00	6.00	13.34	−0.86	−1.38	0.04
441474	MOL001692	Beta-d-Apiose	C1C(C(C(O1)O)O)(CO)O	150.15	−2.17	4.00	5.00	118.53	−1.46	−3.61	0.03
369312	MOL001694	Perillyl alcohol	CC(=C)C1CCC(=CC1)CO	152.26	2.41	1.00	1.00	46.24	1.23	1.39	0.03
5281671	MOL001696	Morusin	CC(=CCC1=C(OC2=C(C1=O)C(=CC3=C2C=CC(O3)(C)C)O)C4=C(C=C(C=C4)O)O)C	420.49	4.94	3.00	6.00	11.52	0.51	−0.22	0.76
6429301	MOL000193	(*Z*)-Caryophyllene	CC1=CCCC(=C)C2CC(C2CC1)(C)C	204.39	4.75	0	0	30.29	1.82	2.15	0.09
442501	MOL000232	(+)-Alpha-terpineol	CC1=CCC(CC1)C(C)(C)O	154.28	2.42	1.00	1.00	46.30	1.28	1.40	0.03
6552009	MOL000244	(+)-Borneol	CC1(C2CCC1(C(C2)O)C)C	154.28	1.98	1.00	1.00	81.80	1.22	1.47	0.05
445858	MOL000360	Ferulic acid	COC1=C(C=CC(=C1)C=CC(=O)O)O	194.20	1.62	2.00	4.00	39.56	0.47	−0.03	0.06
165675	MOL000671	(+)-Menthol	CC1CCC(C(C1)O)C(C)C	156.30	2.78	1.00	1.00	59.33	1.27	1.42	0.03
7041	MOL000077	2,6-Dimethoxyphenol	COC1=C(C(=CC=C1)OC)O	154.18	1.53	1.00	3.00	37.50	1.24	1.32	0.03
5280443	MOL000008	Apigenin	C1=CC(=CC=C1C2=CC(=O)C3=C(C=C(C=C3O2)O)O)O	270.25	2.33	3.00	5.00	23.06	0.43	−0.61	0.21
65575	MOL000875	Cedrol	CC1CCC2C13CCC(C(C3)C2(C)C)(C)O	222.41	3.16	1.00	1.00	16.23	1.35	1.46	0.12

MW, molecular weight; AlogP, partition coefficient between octanol and water; Hdon, hydrogen bond donor count; Hacc, hydrogen bond acceptor count; OB, oral bioavailability; Caco-2, Caco-2 permeability; BBB, blood–brain barrier; DL, drug likeness.

**Table 2:** Topological parameters of 15 potentially active compounds of Herba Patriniae on CRC in the compound–target interaction network.

PubChem CID	Names	Degree	Betweenness Centrality	Closeness Centrality	Average Shortest Path Length
13855373	Asperglaucide	10	0.322	0.350	2.857
21721831	Orotinin	8	0.473	0.389	2.571
10594416	Bolusanthol B	6	0.156	0.273	3.667
5281671	Morusin	6	0.254	0.344	2.905
222284	Sitosterol	5	0.110	0.253	3.952
5280794	Stigmasterol	5	0.110	0.253	3.952
5280863	Kaempferol	4	0.038	0.318	3.143
5280445	Luteolin	4	0.038	0.318	3.143
5280443	Apigenin	4	0.038	0.318	3.143
5280343	Quercetin	4	0.040	0.292	3.429
5280442	Acacetin	3	0.058	0.273	3.667
821366	Sinoacutine	1	0	0.210	4.762
127454	Villosolside	1	0	0.172	5.810
5490819	Villosol	1	0	0.208	4.810
54670067	Vitamin C	1	0	0.210	4.762

**Table 3:** Topological parameters of 28 targets of Herba Patriniae on CRC in the compound–target interaction network.

Symbols	Target Names	Uniprot ID	Degree	Betweenness Centrality	Closeness Centrality	Average Shortest Path Length
GSK3B	Glycogen synthase kinase-3 beta	P49841	6	0.071	0.264	3.786
PTGS2	Cyclooxygenase-2	P35354	6	0.265	0.347	2.881
EGFR	Epidermal growth factor receptor erbB1	P00533	5	0.121	0.326	3.071
AKT1	Serine/threonine-protein kinase AKT	P31749	4	0.005	0.255	3.929
BRAF	Serine/threonine-protein kinase B-raf	P15056	3	0.167	0.341	2.929
PIK3CG	PI3-kinase p110-gamma subunit	P48736	3	0.072	0.347	2.881
PPARG	Peroxisome proliferator-activated receptor gamma	P37231	3	0.285	0.311	3.214
VDR	Vitamin D receptor	P11473	3	0.048	0.207	4.833
MAPK1	MAP kinase ERK2	P28482	2	0.048	0.261	3.833
MAPK8	c-Jun *n*-terminal kinase 1	P45983	2	0.048	0.264	3.786
PIK3CA	PI3-kinase p110-alpha subunit	P42336	2	0.033	0.326	3.071
PIK3CB	PI3-kinase p110-beta subunit	P42338	2	0.033	0.326	3.071
PIK3CD	PI3-kinase p110-delta subunit	O00329	2	0.033	0.326	3.071
PPARD	Peroxisome proliferator-activated receptor delta	Q03181	2	0	0.205	4.881
PTGER2	Prostanoid EP2 receptor (by homology)	P43116	2	0	0.205	4.881
PTGES	Prostaglandin E synthase	O14684	2	0	0.205	4.881
RAF1	Serine/threonine-protein kinase RAF	P04049	2	0.030	0.275	3.643
TYMS	Thymidylate synthase (by homology)	P04818	2	0.060	0.302	3.310
EPHB1	Ephrin type-B receptor 1	P54762	1	0	0.215	4.643
EPHB2	Ephrin type-B receptor 2	P29323	1	0	0.215	4.643
EPHB3	Ephrin type-B receptor 3	P54753	1	0	0.215	4.643
HDAC2	Histone deacetylase 2	Q92769	1	0	0.261	3.833
MAP2K1	Dual specificity mitogen-activated protein kinase 1	Q02750	1	0	0.261	3.833
MAPK10	c-Jun *n*-terminal kinase 3	P53779	1	0	0.258	3.881
MAPK3	MAP kinase ERK1 (by homology)	P27361	1	0	0.258	3.881
PLA2G2A	Phospholipase A2 group IIA	P14555	1	0	0.215	4.643
SFRP1	Secreted frizzled-related protein 1	Q8N474	1	0	0.261	3.833
TGFBR1	TGF-beta receptor type I	P36897	1	0	0.261	3.833

**Table 4:** Topological parameters of 23 interconnected targets of Herba Patriniae on CRC in the PPI network.

Symbols	Degree	Betweenness Centrality	Closeness Centrality	Average Shortest Path Length
PIK3CA	11	0.201	0.595	1.682
AKT1	9	0.111	0.579	1.727
MAP2K1	9	0.157	0.579	1.727
MAPK3	9	0.092	0.579	1.727
PTGS2	9	0.231	0.564	1.773
MAPK1	8	0.043	0.564	1.773
RAF1	8	0.027	0.524	1.909
EGFR	7	0.119	0.564	1.773
PIK3CB	7	0.012	0.500	2.000
PIK3CD	7	0.012	0.500	2.000
MAPK8	6	0.071	0.512	1.955
BRAF	5	0.004	0.489	2.045
PIK3CG	5	0.008	0.478	2.091
GSK3B	4	0	0.449	2.227
EPHB1	4	0.071	0.449	2.227
PPARG	4	0.093	0.458	2.182
EPHB2	3	0.024	0.400	2.500
PTGES	2	0	0.373	2.682
EPHB3	2	0	0.319	3.136
MAPK10	2	0	0.415	2.409
PLA2G2A	2	0	0.407	2.455
PTGER2	2	0	0.373	2.682
HDAC2	1	0	0.319	3.136

**Table 5:** IC_50_ values of human colon cancer cells and normal cells treated with PHW (μg/mL) and 5-fluorouracil (μM). Values are the mean ± SD.

Cell Lines	PHW (μg/mL)		5-Fluorouracil (μM)	
	24 h	48 h	24 h	48 h
HCT116	638.1 ± 52.0	378.1 ± 19.5	>200	14.84 ± 0.31
HT29	>800	751.1 ± 27.5	>200	>200
LoVo	>800	721.7 ± 12.9	>200	85.18 ± 23.51
Hs27	>800	>800	>200	>200

## Data Availability

The data presented in this study are available on request from the corresponding author.

## Supplementary Materials

The following are available online, Figure S1: Full chromatogram of *Patrinia heterophylla* water extract detected by UHPLC-MS. Table S1: Chemical constituents of *Patrinia heterophylla* water extract detected by UHPLC-MS. Table S2. Gene primers sequences. Table S3. Lists of antibodies used for the western blot assays.

## Sample Availability

Not available.
