# Supplementary material for: Integrated Network Pharmacology Analysis and In Vitro Validation Revealed the Potential Active Components and Underlying Mechanistic Pathways of Herba Patriniae in Colorectal Cancer
Source: Molecules. 2021 Oct 5;26(19):6032. doi: 10.3390/molecules26196032 (PMC8513027; doi:10.3390/molecules26196032)
Supplement: Supplementary file 1 [file molecules-26-06032-s001.zip › molecules-1356617-supplementary.pdf]

Integrated Network Pharmacology Analysis and In Vitro Validation Revealed the Potential Active Components and Underlying Mechanistic Pathways of Herba Patriniae in Colorectal Cancer

Huihai Yang [1], Man-Kit Cheung [2], Grace Gar-Lee Yue [1], Ping-Chung Leung [1], Chun-Kwok Wong [1,3], Clara Bik-San Lau [1,4,*]

[1] Institute of Chinese Medicine and State Key Laboratory of Research on Bioactivities and Clinical Applications of Medicinal Plants, The Chinese University of Hong Kong, Shatin, New Territories, Hong Kong

[2] Department of Surgery, The Chinese University of Hong Kong, Shatin, New Territories, Hong Kong

[3] Department of Chemical Pathology, The Chinese University of Hong Kong, Shatin, New Territories, Hong Kong

[4] Li Dak Sum Yip Yio Chin R&D Centre for Chinese Medicine, The Chinese University of Hong Kong, Shatin, New Territories, Hong Kong

Running title: Network pharmacology of the Herba Patriniae (Bai Jiang Cao)

***Correspondence**:

Prof. Clara Bik San Lau

Institute of Chinese Medicine

E305, Science Centre East Block

The Chinese University of Hong Kong

Shatin, New Territories, Hong Kong

Email: claralau@cuhk.edu.hk

ORCID: 0000-0002-7409-1270

Table S1. Chemical constituents of *Patrinia heterophylla* water extract detected by UHPLC-MS

Compounds	Retention times (min)	Molecular weights	Precursors and products
Asperglaucide	11.874	444.57	445→123
Villosolside	3.253	362.42	363→183
Villosol	3.581	200.26	201→109
Ascorbic acid	1.049	176.14	175→59

Table S2. Gene primers sequences

Gene names	Species	Forward primers	Reverse primers
EGFR	Human	TCCCTCAGCCACCCATATGTAC	GTCTCGGGCCATTTTGGAGAATTC
PIK3CA	Human	TGGATGCTCTACAGGGCTTT	GTCTGGGTTCTCCCAATTCA
AKT1	Human	TCTATGGCGCTGAGATTGTG	CTTAATGTGCCCGTCCTTGT
GAPDH	Human	CGAGATCCCTCCAAAATCAA	GGTGCTAAGCAGTTGGTGGT

Table S3. Lists of antibodies used for the western blot assays

Antibodies	Species	Producers	Cat. No.	Dilution (WB)
EGFR	Rabbit	Cell Signaling Technology	4267S	1:1000
p-EGFR	Rabbit	Cell Signaling Technology	3777S	1:1000
PI3K	Rabbit	Cell Signaling Technology	3358S	1:1000
p-PI3K	Rabbit	Cell Signaling Technology	4228S	1:1000
AKT	Rabbit	Cell Signaling Technology	4685S	1:1000
p-AKT	Rabbit	Cell Signaling Technology	4058S	1:1000
Cathepsin B	Rabbit	Cell Signaling Technology	31718S	1:1000
β-actin	Mouse	Sigma	A5316	1:3000
HRP-labeled Goat Anti-Rabbit IgG(H+L)	Goat	Akoya Biosciences	200319028	1:3000
HRP-labeled Goat Anti-Mouse IgG(H+L)	Goat	Akoya Biosciences	200402006	1:3000

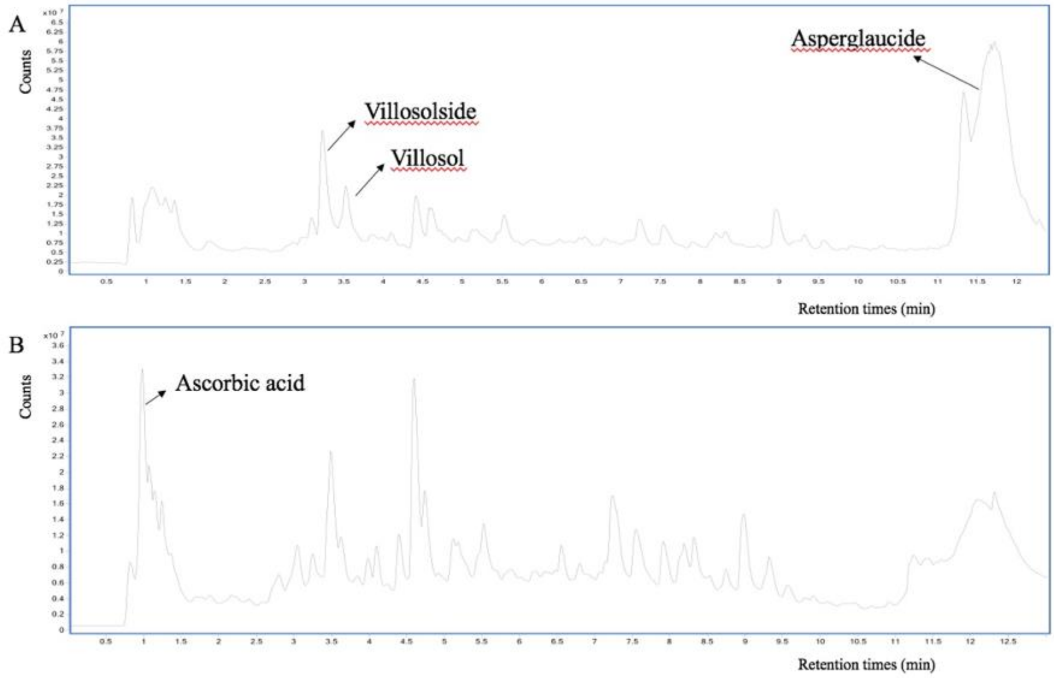

Figure S1. Full chromatogram of *Patrinia heterophylla* water extract detected by UHPLC-MS. (A) positive mode; (B) negative mode.
